# A Hematologic Mimic: Sarcoidosis Presenting With Myelodysplastic Syndrome-Like Marrow Dysplasia, Massive Splenomegaly, and Pancytopenia

**DOI:** 10.7759/cureus.107286

**Published:** 2026-04-18

**Authors:** Shivam Singh, Jaishree Sharma, Deepak Sharma, Anurag Prasad, Shrey Srivastav

**Affiliations:** 1 Department of General Medicine, School of Medical Sciences and Research Sharda University, Greater Noida, IND

**Keywords:** bone marrow dysplasia, fdg pet-ct, granulomatous disease, hypersplenism, massive splenomegaly, myelodysplastic syndrome, pancytopenia, parathyroid hormone–independent hypercalcemia, sarcoidosis

## Abstract

Sarcoidosis is a multisystem granulomatous disorder of unknown etiology with heterogeneous clinical manifestations and significant diagnostic challenges, particularly when extrapulmonary involvement predominates. Although splenic, hematologic, metabolic, and lymphatic manifestations are individually recognized, their simultaneous occurrence is exceptionally rare. We report a diagnostically complex case of sarcoidosis presenting with massive splenomegaly, pancytopenia, parathyroid hormone-independent hypercalcemia with renal dysfunction, and bone marrow findings suggestive of myelodysplastic syndrome.

A 53-year-old man was incidentally found to have pancytopenia during routine evaluation and was asymptomatic at presentation, without constitutional symptoms. Abdominal imaging revealed massive splenomegaly with preserved hepatic morphology. Bone marrow aspiration and biopsy demonstrated multilineage dysplasia with low blast percentage, favoring myelodysplastic neoplasia; however, this diagnosis was discordant with the presence of massive splenomegaly. Subsequent biochemical evaluation revealed hypercalcemia with suppressed parathyroid hormone levels and associated renal dysfunction, prompting further investigation. ^18^F-fluorodeoxyglucose positron emission tomography-computed tomography demonstrated extensive FDG-avid mediastinal, hilar, cervical, and abdominal lymphadenopathy with gross splenomegaly, a pattern suggestive of granulomatous disease. Elevated serum angiotensin-converting enzyme levels supported this impression. Definitive diagnosis was established by excisional cervical lymph node biopsy, which revealed non-caseating granulomatous lymphadenitis consistent with sarcoidosis.

This case illustrates a rare and misleading presentation of sarcoidosis that closely mimicked a primary hematologic disorder. It underscores the importance of maintaining a broad differential diagnosis and emphasizes the critical role of integrated clinicopathologic correlation and tissue confirmation in patients presenting with unexplained cytopenias and massive splenomegaly.

## Introduction

Sarcoidosis is a systemic granulomatous disorder of unknown cause, defined histopathologically by non-caseating epithelioid cell granulomas and characterized by heterogeneous clinical presentations ranging from incidental radiographic abnormalities to progressive multisystem disease [1,2]. The disease most frequently involves the lungs and intrathoracic lymph nodes, but clinically important extrapulmonary involvement is well-recognized and may affect the skin, eyes, liver, spleen, bone marrow, kidneys, and calcium metabolism, creating substantial diagnostic complexity when presentations fall outside the classic pulmonary phenotype [1-3]. Because sarcoidosis can mimic infection, malignancy, and immune-mediated disorders, contemporary diagnostic frameworks emphasize compatible clinical-radiologic features plus tissue confirmation of non-caseating granulomas, with careful exclusion of alternative granulomatous conditions [1,2].

Extrapulmonary sarcoidosis may present through organ-specific syndromes or systemic metabolic derangements. Renal and calcium disturbances are clinically important because granulomatous macrophage activation can increase extrarenal 1-α hydroxylase activity, leading to excess calcitriol and parathyroid hormone-independent hypercalcemia, which may precipitate or worsen renal dysfunction [2,3]. Separately, hematologic abnormalities may occur through multiple mechanisms, including chronic inflammation, hypersplenism, and, less commonly, granulomatous bone marrow involvement, and these patterns can closely resemble hematologic malignancy or bone marrow failure syndromes in initial evaluation [1-3].

Advanced imaging can help define the extent and activity of disease and guide optimal biopsy sites. In particular, 18F-FDG PET/CT has an established role in selected patients, especially when conventional investigations are inconclusive, by identifying metabolically active inflammatory foci, mapping multisystem involvement, and helping target accessible, high-yield tissue for histopathology [4]. However, FDG-avid lymphadenopathy and splenic involvement may still be radiologically indistinguishable from lymphoma or disseminated malignancy, reinforcing that histopathological confirmation remains the diagnostic cornerstone [1-4]. In this context, we report an unusual case of sarcoidosis presenting with a rare convergence of massive splenomegaly, pancytopenia, parathyroid hormone-independent hypercalcemia with renal dysfunction, and diffuse FDG-avid lymphadenopathy, initially mimicking a primary hematologic disorder.

## Case presentation

A 53-year-old man was evaluated in the outpatient clinic following the incidental detection of pancytopenia on an outside complete blood count. At presentation, he was entirely asymptomatic and specifically denied fever, weight loss, anorexia, night sweats, or other constitutional symptoms. His past medical history was notable only for type 2 diabetes mellitus of approximately 10-12 years’ duration. There was no history of chronic alcohol consumption.

The initial trigger for further evaluation was an abdominal ultrasound performed outside, which revealed massive splenomegaly measuring approximately 22 cm with preserved liver echotexture and normal renal size. This unexpected finding prompted referral for evaluation of splenomegaly in the setting of cytopenias.

**Figure 1:**
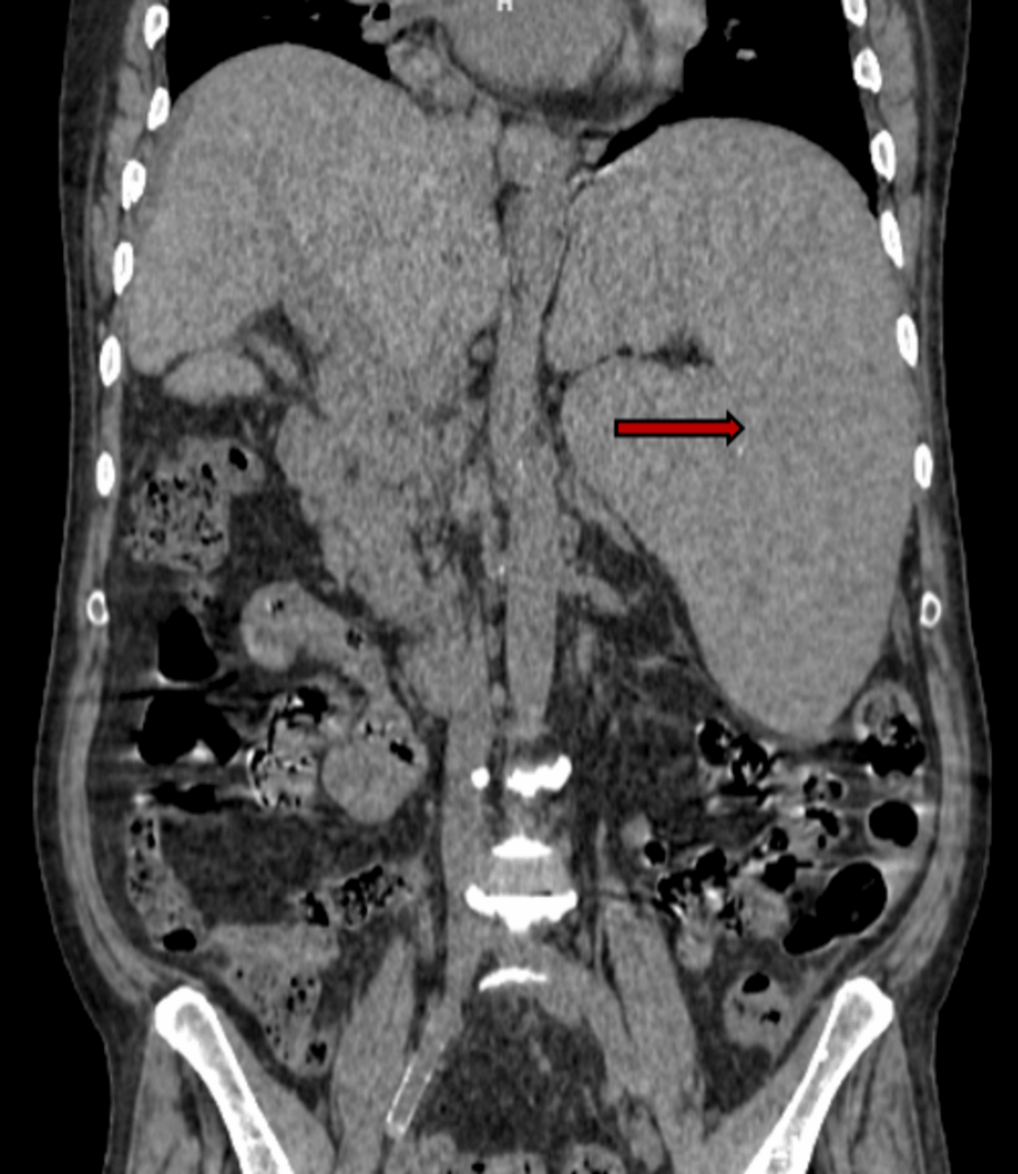
Non-contrast CT scan of the abdomen showing gross splenomegaly The red arrow shows the enlarged spleen.

A subsequent non-contrast CT scan of the whole abdomen confirmed gross splenomegaly with dilatation of the portal vein, multiple perisplenic and periportal collateral vessels, and minimal ascites (Figure 1). The liver was reported as normal in size and morphology, without focal lesions or intrahepatic biliary dilatation. The scan also identified a few enlarged lymph nodes in the peripancreatic, mesenteric, and retroperitoneal regions, as well as supradiaphragmatic lymph nodes. Non-contrast CT scan findings of the whole abdomen are given in Figure 1.

Repeat in-house hematological evaluation confirmed pancytopenia with decreased hemoglobin, leukocyte count, and platelet count (Table 1). Red cell indices were normocytic with increased anisocytosis. Findings of laboratory investigations at presentation and follow-up are summarized in Table 1.

**Table 1:** Summary of laboratory investigations at presentation and follow-up RDW-CV, red cell distribution width-coefficient of variation; RDW-SD, red cell distribution width-standard deviation; AST, aspartate aminotransferase; ALT, alanine aminotransferase; ACE, angiotensin-converting enzyme; NA, not available

Parameter	At Presentation	At Follow-up	Reference Range	Interpretation
Hemoglobin (g/dL)	6.3	8.8	13–17	Improved
Total leukocyte count (×10³/µL)	2.93	5.7	4–11	Improved
Platelet count (×10³/µL)	50	138	150–400	Improved
Mean corpuscular volume (fL)	94.5	87.5	80–100	Normal
RDW-CV (%)	17	17.3	11.5–14.5	Increased
RDW-SD (fL)	59.5	57.8	39–46	Increased
Absolute neutrophil count (×10³/µL)	2.1	3.88	1.5–7.5	Normal
Absolute lymphocyte count (×10³/µL)	0.47	1.31	1.0–4.0	Improved
Serum calcium (mg/dL)	11.4	8.9	8.5–10.5	Normalized
Blood urea (mg/dL)	76	72	10–40	Improved
Serum creatinine (mg/dL)	3.4	2.2	0.7–1.2	Improved
Sodium (mmol/L)	138	137	135–145	Normal
Potassium (mmol/L)	5.1	4.3	3.5–5.0	Normalized
Chloride (mmol/L)	104	103	98–107	Normal
AST (U/L)	21	34	10–40	Normal
ALT (U/L)	12	36	7–56	Normal
Alkaline phosphatase (U/L)	251	88	44–147	Improved
Gamma-glutamyl transferase (U/L)	73	100	9–48	Elevated
Albumin (g/dL)	2.3	4.4	3.5–5.0	Improved
Total protein (g/dL)	6.1	7	6.0–8.3	Normal
Albumin/Globulin ratio	0.6	1.7	1.0–2.0	Improved
Parathyroid hormone	2.4 pg/mL	NA	15–65 pg/mL	NA
Serum ACE	75.96 U/L	NA	8–52 U/L	NA

Given the combination of pancytopenia and marked splenomegaly, bone marrow aspiration and trephine biopsy were performed early in the diagnostic evaluation. The marrow was reported as normocellular for age, with dysplastic changes involving multiple lineages, including dyserythropoiesis with megaloblastoid change and binucleation, and dysmegakaryopoiesis characterized by hypolobated forms and occasional micromegakaryocytes. Blasts constituted a low percentage of the aspirate differential, and megakaryocytes were noted to be reduced on aspirate. Importantly, no granulomas or metastatic deposits were identified. The overall impression favored myelodysplastic neoplasia (low blasts), with a recommendation for clinical correlation, exclusion of secondary causes of dysplasia, and further cytogenetic and molecular evaluation.

However, at this stage, a key clinicoradiological discordance was evident, as massive splenomegaly is atypical for myelodysplastic neoplasia alone, prompting continued investigation for an additional or alternative systemic process.

Biochemical evaluation subsequently revealed hypercalcemia with renal dysfunction (Table 1). In view of hypoalbuminemia (albumin 2.3 g/dL), corrected serum calcium was calculated and found to be 12.76 mg/dL, indicating significant hypercalcemia. Liver biochemistry demonstrated preserved transaminases with a cholestatic pattern and hypoalbuminemia. Given that hypercalcemia could not be readily explained by marrow findings alone, a structured evaluation for parathyroid hormone-independent hypercalcemia was undertaken. Parathyroid hormone levels were suppressed, supporting a non-parathyroid hormone (PTH)-mediated mechanism.

The differential diagnosis at this stage included plasma cell dyscrasia, lymphoma or metastatic malignancy, and granulomatous disease such as sarcoidosis. Multiple myeloma was subsequently excluded, while lymphoma remained a consideration in view of splenomegaly and cytopenias despite the absence of constitutional symptoms.

To further evaluate for occult malignancy or inflammatory disease, an 18F-fluorodeoxyglucose positron emission tomography-computed tomography (18F-FDG PET-CT) was performed in the clinical context of suspected myelodysplastic neoplasia.

**Figure 2:**
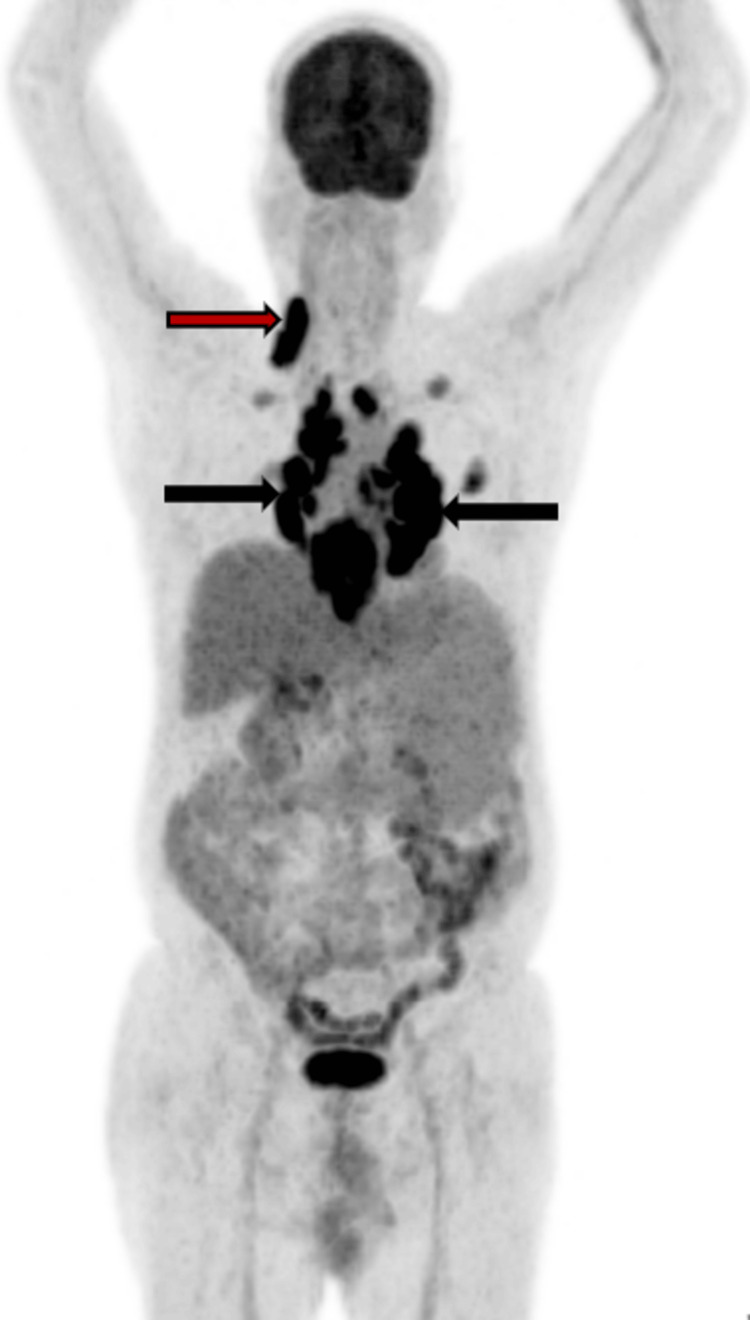
Whole-body ¹⁸F-fluorodeoxyglucose (FDG) positron emission tomography (PET) maximum intensity projection image demonstrating intense FDG uptake in upper right cervical lymph nodes and bilateral hilar lymph nodes as shown by the red and black arrows, respectively

The whole-body maximum intensity projection PET image is shown in Figure 2.

**Figure 3:**
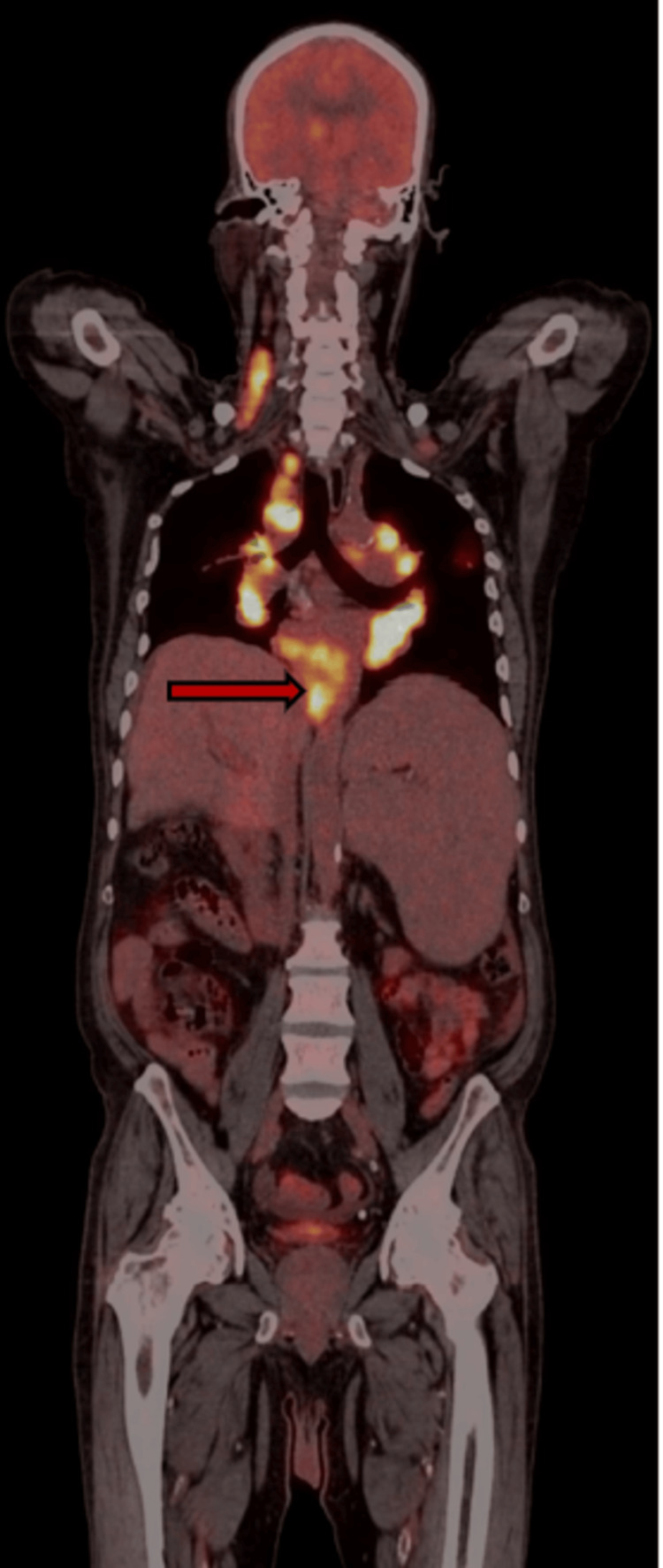
Coronal fused ¹⁸F-fluorodeoxyglucose (FDG) positron emission tomography (PET)-computed tomography (CT) image showing hypermetabolic lymphadenopathy involving paraesophageal nodal stations as shown by the red arrow No focal FDG-avid lesions suggestive of a primary malignant mass are identified.

Coronal fused PET-CT imaging is shown in Figure 3.

**Figure 4:**
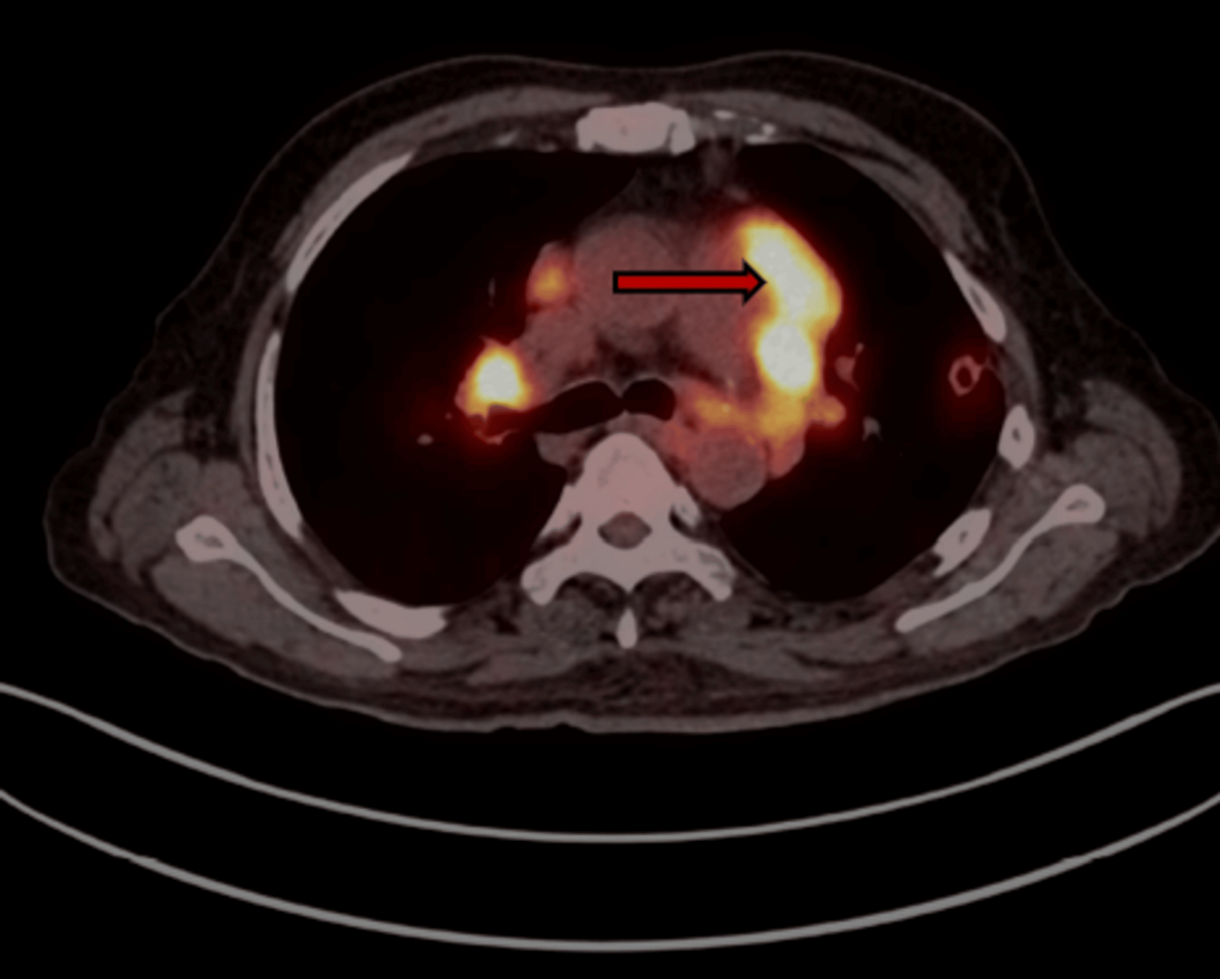
Axial fused ¹⁸F-fluorodeoxyglucose (FDG) positron emission tomography (PET)-computed tomography (CT) image at the level of the mediastinum demonstrating enlarged FDG-avid prevascular lymph nodes as shown by the red arrow

Axial fused PET-CT imaging is shown in Figure 4.

The study demonstrated extensive FDG-avid lymphadenopathy predominantly involving mediastinal and hilar stations, with additional cervical and upper abdominal lymphadenopathy. The spleen demonstrated physiological FDG uptake but remained markedly enlarged. Overall, the imaging pattern favored granulomatous disease such as sarcoidosis, and histopathological confirmation was recommended.

In support of this impression, serum angiotensin-converting enzyme levels were elevated (Table 1).

Given the presence of accessible cervical lymphadenopathy, an excisional biopsy of a right cervical lymph node was performed. Histopathological examination revealed lymphoid tissue replaced by compact non-caseating granulomas composed of epithelioid histiocytes and occasional multinucleated giant cells without central necrosis.

**Figure 5:**
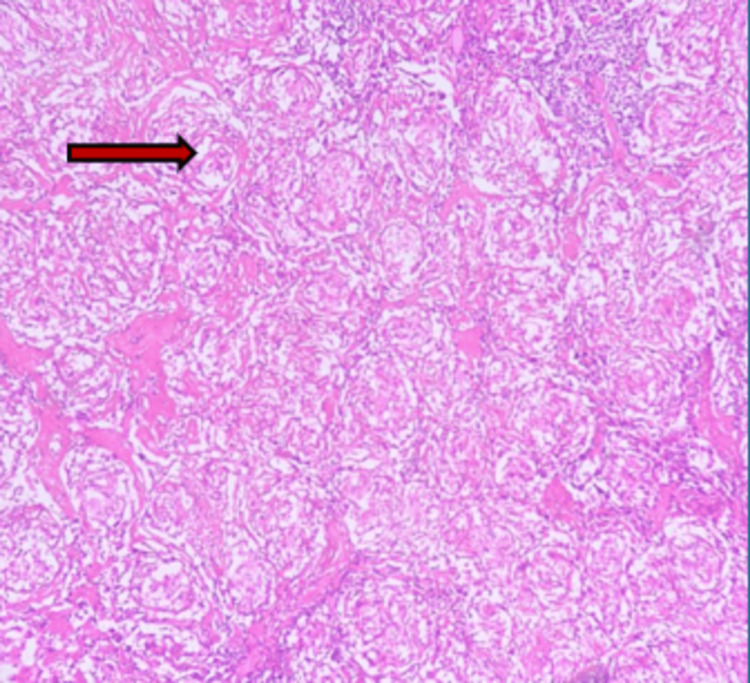
Hematoxylin and eosin–stained section of the excised cervical lymph node showing near-complete effacement of normal nodal architecture by multiple well-formed, compact, non-caseating epithelioid cell granulomas. The granulomas are composed predominantly of epithelioid histiocytes with occasional multinucleated giant cells, without central necrosis. No features of malignancy are identified (H&E stain, ×100). The red arrow shows compact non-caseating granulomas.

Histopathological findings are shown in Figure 5.

Ziehl-Neelsen staining was non-contributory. The final impression was non-caseating granulomatous lymphadenitis consistent with sarcoidosis.

Taken together, the patient’s evaluation revealed a distinctive constellation of findings: asymptomatic pancytopenia with massive splenomegaly, parathyroid hormone-independent hypercalcemia with renal dysfunction, bone marrow dysplasia suggestive of myelodysplastic neoplasia without granulomatous infiltration, and diffuse FDG-avid lymphadenopathy, with definitive tissue confirmation of sarcoidosis on lymph node biopsy.

The patient was initiated on oral prednisolone at a dose of 60 mg/day, followed by gradual tapering based on clinical and biochemical response. Close biochemical and hematologic monitoring was undertaken. Serum calcium levels normalized within two weeks of therapy initiation, accompanied by progressive improvement in renal function parameters. Hemoglobin and platelet counts demonstrated gradual improvement over follow-up, consistent with resolution of hypersplenism-related cytopenias (Table 1). The patient remained clinically stable and continues tapering corticosteroid therapy under multidisciplinary follow-up.

## Discussion

Sarcoidosis is a multisystem inflammatory disease of unknown etiology characterized by the formation of non-caseating granulomas, most frequently involving the lungs and intrathoracic lymph nodes [5]. Although pulmonary involvement predominates, extrapulmonary manifestations are well-recognized and may involve the lymphatic system, spleen, bone marrow, kidneys, and calcium metabolism, often leading to diagnostic confusion when presentations mimic malignancy or hematologic disorders [5].

Splenic involvement in sarcoidosis is common histologically but is usually mild and clinically silent. Clinically detectable splenomegaly occurs in approximately 10% of patients, whereas massive or giant splenomegaly is rare, reported in only about 3% of cases [6]. Fordice et al. highlighted that massive splenomegaly due to sarcoidosis is uncommon and frequently misinterpreted as lymphoma, often leading to surgical exploration [6]. Subsequent reports have reinforced this observation, documenting sarcoidosis presenting primarily with massive splenomegaly and cytopenias, thereby closely mimicking hematologic malignancies [7-10].

Cytopenias in sarcoidosis are multifactorial and may result from hypersplenism, granulomatous infiltration of the bone marrow, immune-mediated mechanisms, or chronic inflammation [11]. Pancytopenia is uncommon but has been reported in association with extensive splenic involvement and bone marrow disease [8-10]. Thadani et al. described pancytopenia secondary to massive splenomegaly with hematologic remission following splenectomy, underscoring hypersplenism as an important mechanism [8]. Similarly, Stoelting et al. and Saito et al. reported severe cytopenias associated with giant splenomegaly that initially prompted evaluation for hematologic malignancy [7,9].

Bone marrow involvement in sarcoidosis is rare, with reported prevalence ranging from 0.3% to 2.2%, and is often clinically unsuspected [11]. When present, granulomatous inflammation of the marrow can produce cytopenias and morphologic abnormalities that may be misinterpreted as myelodysplastic syndrome [11-13]. Paul et al. reported sarcoidosis presenting with pancytopenia and marrow granulomas, initially raising concern for marrow failure syndromes [12]. Gubatan et al. emphasized that sarcoidosis can masquerade as clonal hematologic disease, as granulomatous inflammation may produce dysplastic-appearing changes without evidence of true myeloid neoplasia [13]. In contrast, Tunkel et al. described the development of cytogenetically confirmed 5q- myelodysplastic syndrome in a patient with sarcoidosis, representing a true coexistence rather than a mimicking phenomenon [14]. In contrast, Airaghi et al. described sarcoidosis occurring in a patient with established 5q-myelodysplastic syndrome, suggesting that immune dysregulation may predispose to sarcoid granuloma formation rather than a direct causal relationship [15].

Hypercalcemia is a well-recognized extrapulmonary manifestation of sarcoidosis and results from increased extrarenal production of 1,25-dihydroxyvitamin D by activated macrophages within granulomas, leading to parathyroid hormone-independent hypercalcemia [16]. This metabolic abnormality may be complicated by renal dysfunction. Several case reports have described sarcoidosis presenting with hypercalcemia, acute kidney injury, and cytopenias, thereby mimicking plasma cell dyscrasias or metastatic malignancy [12,17,18]. Saba et al. and Kechaou et al. reported patients in whom hypercalcemia and renal impairment were the dominant presenting features, with malignancy initially suspected and later excluded following histopathological confirmation [17,18].

Advanced imaging modalities, such as 18F-FDG PET-CT, are useful for detecting multisystem involvement and guiding biopsy in sarcoidosis; however, diffuse FDG-avid lymphadenopathy involving mediastinal, hilar, cervical, and abdominal nodes may closely resemble lymphoma [19]. In the present case, the absence of a focal primary mass, the pattern of widespread symmetric lymphadenopathy, and associated biochemical findings favored a granulomatous process over lymphoma. Albert et al. and Kruithoff et al. emphasized that extensive FDG-avid lymphadenopathy with splenic enlargement should prompt consideration of sarcoidosis in the appropriate clinical context, particularly in the absence of constitutional symptoms [19,20]. Ultimately, histopathological confirmation remains essential for definitive diagnosis.

Previously reported cases are summarized in Table 2.

**Table 2:** Reported cases of sarcoidosis presenting with splenomegaly, cytopenias, hypercalcemia, and/or bone marrow involvement Hb, hemoglobin; Plt, platelet count; Ca, calcium; Cr, creatinine; AKI, acute kidney injury; MDS, myelodysplastic syndrome; PTH, parathyroid hormone; LN, lymph node; FDG, fluorodeoxyglucose; PET-CT, positron emission tomography–computed tomography; NR, not reported

Study	Age/Sex	Key Presentation	Splenic Findings	Cytopenias	Hypercalcemia / Renal	Lymphadenopathy	Bone Marrow Findings	Tissue Confirmation	Treatment / Outcome
Fordice et al., 1992 [6]	NR	Massive splenomegaly, suspected lymphoma	Spleen 2250 g (one of the largest reported)	NR	NR	NR	Not described	Splenic sarcoidosis (intra-operative)	Diagnostic splenectomy; outcome NR
Stoelting et al., 2020 [7]	58/F	Epistaxis, severe thrombocytopenia	25 cm; 2875 g; splenic artery aneurysms	Hb 4.8 g/dL; Plt 6,000/µL	Absent	Absent	Non-diagnostic; no malignancy	Splenic granulomas	Embolization → splenectomy; recovery
Thadani et al., 1975 [8]	53/M	Abdominal discomfort, pancytopenia	17 × 15 × 8 cm; 1250 g	Pancytopenia + hemolysis	Absent	Absent clinically	Normal cellularity	Liver + spleen granulomas	Splenectomy → initial improvement; later hemolytic crisis
Saito et al., 2020 [9]	22/F	Abdominal distension, fatigue	13 × 24 cm; 4300 g	Severe pancytopenia	Calcium normal; renal dysfunction (compression)	Mediastinal + cervical	Hypercellular; no malignancy	Liver, spleen, skin, lung granulomas	Splenectomy → hematologic & renal improvement
Saad et al., 2024 [10]	28/M	Fatigue, night sweats	46 × 20 cm; PET-avid	Thrombocytopenia	Absent	Absent	Normal	Splenic granulomas	Splenectomy → asymptomatic at 2 years
Paul et al., 2014 [12]	65/M	Functional decline, weight loss	Splenomegaly 20.7 cm	Pancytopenia	Ca 10.9 mg/dL; PTH suppressed	Absent	Granulomas present	Bone marrow + liver	Diagnostic clarification; malignancy excluded
Gubatan et al., 2016 [13]	57/F	Confusion, polydipsia	Absent	Mild pancytopenia	Ca 14.4 mg/dL; AKI	Absent	Granulomas only	Bone marrow	Steroids → normalization
Tunkel et al., 1990 [14]	NR	Sarcoidosis with MDS	Mild / NR	Anemia	Absent	Present	True 5q– MDS	Lung + liver	Descriptive association
Airaghi et al., 2001 [15]	56/F	Sarcoidosis in known 5q– MDS	Not reported	Not reported	Not reported	Not reported	True 5q– MDS	Lung + skin	Cytokine imbalance hypothesis
Saba et al., 2016 [17]	72/F	Fatigue, dizziness	Splenomegaly 23 cm	Pancytopenia	Ca 14 mg/dL; AKI	Mediastinal + axillary	Granulomatous marrow	Bone marrow	Steroids → Ca & renal recovery
Kechaou et al., 2025 [18]	43/F	Prolonged fever, weight loss	23 → 26 cm	Pancytopenia	Ca ~13 mg/dL; renal normal	Mediastinal + abdominal	Not described	LN + skin granulomas	Steroids → complete regression
Albert et al., 2015 [19]	28/M	Fatigue, abdominal pain, weight loss	2022 g spleen	Pancytopenia	Ca 12.7 mg/dL; Cr 2.9 mg/dL	Portacaval	No malignancy	Liver + spleen granulomas	Splenectomy + steroids → methotrexate
Kruithoff et al., 1993 [20]	37/M	Pancytopenia, severe hypercalcemia	Massive; 1825 g	Pancytopenia	Ca 16.6 mg/dL; AKI	Mediastinal + gastrohepatic	Mild hypocellularity	Liver + spleen	Splenectomy → rapid normalization
Present Case	53/M	Asymptomatic pancytopenia	~22 cm (massive)	Pancytopenia	Hypercalcemia with AKI; PTH suppressed	Diffuse FDG-avid (mediastinal, hilar, cervical, abdominal)	MDS-like dysplasia (non-clonal)	LN biopsy: non-caseating granulomas	Sarcoidosis diagnosed; steroids → hypercalcemia resolved

The present case represents a rare and diagnostically challenging constellation of sarcoidosis manifestations. The patient presented incidentally with pancytopenia and massive splenomegaly, initially suggesting a primary hematologic disorder. Bone marrow aspiration and biopsy demonstrated features suggestive of myelodysplastic syndrome, a diagnosis that does not explain massive splenomegaly. Subsequent identification of parathyroid hormone-independent hypercalcemia with renal dysfunction, elevated serum angiotensin-converting enzyme levels, and widespread FDG-avid lymphadenopathy redirected evaluation toward granulomatous disease. Definitive excisional lymph node biopsy, revealing non-caseating granulomas, established the diagnosis of sarcoidosis.

Compared with previously published reports, this case uniquely combines massive splenomegaly with hypersplenism, pancytopenia with marrow findings mimicking myelodysplastic syndrome, parathyroid hormone-independent hypercalcemia with renal impairment, and diffuse FDG-avid lymphadenopathy within a single patient [6-20]. While individual components of this presentation have been reported separately, their simultaneous occurrence is exceedingly rare. This case underscores the importance of comprehensive clinicopathologic correlation and tissue diagnosis before assigning a diagnosis of primary hematologic malignancy or clonal marrow disorder. Early recognition of this rare presentation can prevent misdiagnosis and inappropriate treatment, reinforcing the need for a multidisciplinary approach in complex systemic diseases.

The simultaneous occurrence of massive splenomegaly, pancytopenia, hypercalcemia, and myelodysplastic syndrome-like marrow dysplasia in a single patient represents an exceptionally rare and diagnostically challenging presentation of sarcoidosis.

This case has certain limitations. Advanced cytogenetic and molecular studies were not performed to definitively exclude clonal myeloid neoplasia. Additionally, measurement of 1,25-dihydroxyvitamin D levels was not available. As a single case report, the findings may not be generalizable. Furthermore, distinguishing sarcoidosis from hematologic malignancies can be challenging, particularly in the presence of bone marrow dysplasia.

## Conclusions

Sarcoidosis can rarely present with a complex constellation of extrapulmonary manifestations that closely mimic primary hematologic disorders. This case highlights an unusual presentation characterized by massive splenomegaly, pancytopenia, parathyroid hormone-independent hypercalcemia with renal dysfunction, and bone marrow findings suggestive of myelodysplastic syndrome.

Definitive diagnosis was achieved only through integrated clinical evaluation and tissue confirmation demonstrating non-caseating granulomatous disease. Awareness of such atypical presentations is essential to avoid misdiagnosis and inappropriate management. Sarcoidosis should be considered in the differential diagnosis of unexplained cytopenias and splenomegaly, even when initial bone marrow findings suggest myelodysplasia, reinforcing the critical importance of comprehensive clinicopathologic correlation. This case also highlights the importance of recognizing diagnostic limitations and maintaining a high index of suspicion to avoid misdiagnosis in complex presentations.
